# Alternative forms of hydration in patients with cancer in the last days of life: study protocol for a randomised controlled trial

**DOI:** 10.1186/s13063-015-0988-3

**Published:** 2015-10-14

**Authors:** Andrew Davies, Melanie Waghorn, Julia Boyle, Ann Gallagher, Sigurd Johnsen

**Affiliations:** Royal Surrey County Hospital, Guildford, Surrey GU2 7XX UK; Surrey CTU, Surrey Clinical Research Centre, School of Biosciences and Medicine, Faculty of Health and Medical Sciences, University of Surrey, Egerton Road, Guildford, Surrey GU2 7XH UK; School of Health Sciences, Faculty of Health and Medical Sciences, University of Surrey, Guildford, Surrey GU2 5XH UK

**Keywords:** End-of-life care, Clinically assisted hydration, Palliative care

## Abstract

**Background:**

The provision of clinically assisted hydration at the end of life is one of the most contentious issues in medicine, and indeed within the general population. The reasons for contention include: a) the lack of evidence for or against; b) the disparate opinions of healthcare professionals; and c) the generally positive opinions of patients and their carers about clinically assisted hydration.

**Methods/design:**

The study is a cluster randomised trial to assess the feasibility of conducting an adequately powered, randomised controlled trial of clinically assisted hydration in patients with cancer in the last days of life. Twelve sites, four National Health Service (NHS) hospitals and eight NHS/voluntary sector hospices in the United Kingdom, will be randomised to give either standard intervention A: continuance of oral intake and regular mouth care, or standard intervention B: continuance of oral intake, regular mouth care and clinically assisted hydration. Patients will be included if they: i) have a diagnosis of cancer; ii) are aged ≥ 18 yr; iii) have an estimated prognosis of ≤ 1 week and iv) are unable to maintain sufficient oral intake (1 L per day, measured/estimated); and v) are able to give informed consent. Patients will be excluded if they have contra-indications to receiving clinically assisted hydration. The primary endpoint of interest is the frequency of hyperactive delirium (‘terminal agitation‘), and this will be assessed using the Modified Richmond Agitation and Sedation Scale (administered every four hours). Other data to be collected include the frequency of pain, respiratory secretions (‘death rattle‘), dyspnoea, nausea and vomiting, adverse effects to clinically assisted hydration and overall survival. In addition, data will be collected on the use of anti-psychotic drugs, sedative drugs, analgesics, anti-secretory drugs and other end-of-life medication. The study has obtained full ethical approval.

**Discussion:**

A randomised controlled trial of clinically assisted hydration in end-of-life care is urgently required. This feasibility study will allow methodological and ethical issues to be understood and addressed to ensure that a robust, adequately powered, randomised controlled trial is designed.

**Trial registration:**

ClinicalTrials.gov NCT02344927 (registered 4 June 2014).

**Electronic supplementary material:**

The online version of this article (doi:10.1186/s13063-015-0988-3) contains supplementary material, which is available to authorized users.

## Background

The provision of clinically assisted hydration (CAH) at the end of life is one of the most contentious issues in medicine [1, 2], and indeed within the general population [3, 4]. The reasons for contention include: a) the lack of evidence for/against CAH [5, 6]; b) the disparate opinions of healthcare professionals about CAH [7]; and c) the generally positive opinions of patients and their carers about CAH (and the generally negative opinions about withholding/withdrawing CAH) [7]. It is, therefore, unsurprising that the provision of CAH at the end of life is extremely variable within clinical practice (12–88 % of patients with cancer in the last week of life) [8]. It should be noted that, in this instance, CAH refers to the medical provision of parenteral fluids (intravenous, subcutaneous), and not to the medical provision of enteral fluids (such as administering fluids via a gastrostomy/jejunostomy).

**Table 1:** Standard treatment B: volume of fluid to be administered

Patient’s weight	Volume of fluid
<45 kg	1 L/24 h
45–60 kg	1.5 L/24 h
>60 kg	2 L/24 h

**Table 2:** Overview of study assessments

Assessments	Screening	Treatment period (daily assessments)	End of study
Inclusion/exclusion criteria	X		
Consent	X		
Demographic data	X		
Cancer diagnosis	X		
Concurrent medical history	X		
Regular medication	X	X	
Oral intake	X	X	
Urinary continence	X	X	
4 hourly Modified Richmond Agitation - Sedation Scale scores		X	
4 hourly symptom occurrences		X	
As required medication		X	
Oral intake		X	
Urinary continence		X	
Parenteral fluids administered		X	
Adverse events		X	
Cannulation problems		X	
Participant outcome			X

In 2012, there were 499,331 deaths registered in England and Wales, with cancer being the most common underlying cause of death (29 % total deaths) [9]. There is limited data on the use of CAH in hospitals, and no data on the use of CAH at home or in hospices (although the impression is that CAH is generally not available in the home setting, and is generally not utilised in the hospice setting).

The lack of provision of fluids at the end of life was one of the major issues raised in the recent review of the Liverpool Care Pathway for the Dying Patient (LCP) (which has resulted in the planned withdrawal of the LCP in the United Kingdom) [10]. Indeed, the review notes that “most of the submissions to the Review from relatives and carers that were critical of the LCP made reference to hydration and nutrition”. The review comments that “if fluids are stopped without review over many days, death from dehydration will be inevitable, the lack of hydration having accelerated the dying process”. However, it also highlights that “a systematic review of all of the literature and studies evaluating the benefits of clinically assisted hydration in palliative care patients shows no clear benefit to either length or quality of life”. Of note, the aforementioned systematic review concluded that “more evidence is needed, particularly in relation to effects of clinically-assisted hydration in patients suffering symptoms that might be strongly influenced by hydration (e.g. delirium, and symptoms of fluid overload)” [6].

In 2008, Good et al. conducted a Cochrane systematic review of medically assisted hydration for adult palliative care patients, and concluded that “currently, there are insufficient good quality studies to make any recommendations for practice with regards to the use of medically assisted hydration in palliative care patients” [5]. Good et al. identified five relevant studies [11–15], although only two studies were randomised controlled studies [13, 14]. However, neither of the randomised controlled studies addressed the specific issue of the routine use of CAH at the end of life. Thus, Cerchietti et al. included patients with evidence of dehydration (and/or renal failure), and patients were given relatively low volumes of fluid (1 L per day), and the fluids were only given for 48 h (and not continued until death) [13]. Similarly, Bruera et al. only included patients with evidence of dehydration, and patients were given relatively low volumes of fluid (1 L per day), and the fluids were only continued for 48 h (and not continued until death) [14]. It should be noted that Good et al. re-searched the literature in early 2011, and although they did not find any new studies, they did identify an ongoing randomised controlled trial [16].

In 2013, Parry et al. conducted a rapid evidence review of the literature on pathways focussed on the dying phase in end-of-life care and their key components, and concluded that “the current research evidence base is not sufficient to inform specific recommendations to use or not to use clinically-assisted nutrition and/or hydration” [6]. The review included the new randomised controlled trial [16], which again did not address the specific issue of the routine use of CAH at the end of life. Thus, Bruera et al. only included patients with evidence of dehydration, and patients were given relatively low doses of fluid (1 L per day), and the fluids were continued for a variable duration, that is, “until the patient was unresponsive, developed progressive coma, or died”. The conclusion of this study was that “hydration at 1 L per day did not improve symptoms, quality of life, or survival”.

The purported positive effects of CAH include the maintenance of patient comfort (for example, prevention of thirst, prevention of dry mouth), and the maintenance of renal perfusion/prevention of accumulation of toxins and drugs (prevention of delirium, prevention of opioid toxicity) [17]. In contrast, the purported negative effects of CAH include problems due to fluid overload (for example, worsening of peripheral oedema, worsening of cardiac failure), and problems due to fluid-related complications (for example, worsening of vomiting, worsening of respiratory secretions) [17]. In addition, it has been claimed that ketones and other by-products of dehydration can have positive effects on the patients’ condition/symptom control (analgesic effects, sedative effects). As intimated above, there is little evidence to support/refute these effects in the general population.

Patients with cancer may develop a range of problems in the last days of life, including delirium (’terminal agitation’/’terminal restlessness’ [18]), excess respiratory secretions (’death rattle’), urinary retention or urinary incontinence, and continuance or exacerbation of other symptoms (for example, pain, dyspnoea, nausea and vomiting) [19].

Delirium is one of the most common problems (25–85 % of patients) [20], and one of the most distressing problems (for patients, relatives and healthcare professionals) [21], encountered at the end of life. Dehydration is a recognised cause of delirium, and rehydration a recommended intervention for delirium (in appropriate situations) [20]. Nevertheless, the mainstay of the management of delirium is the use of antipsychotic and/or sedative drugs [20]. Such agents are used in approximately 50 % of patients in the last week of life [22], and although they are generally very effective, they are often associated with untoward sedation (which necessarily impacts the dying process, especially in terms of interpersonal communication). It should be noted that the use of sedative drugs was another major issue raised in the recent review of the LCP (which has resulted in the planned withdrawal of the LCP in the United Kingdom) [10].

### Study rationale and aim

The authors of the Cochrane systematic review discussed the need for further studies in this area, and highlighted the barriers to conducting such studies, including obtaining informed consent, recruitment of subjects, retention of subjects, and presence of confounders [5, 23]. Our aim is to undertake a cluster randomised trial of clinically assisted hydration in non-dehydrated cancer patients at the end of life (that is, in the last week of life) in order to fully assess the practical and ethical issues that need to be addressed in a randomised controlled trial (RCT) and to provide data to adequately power the RCT.

## Methods

### Trial design and setting

The study will be a multi-centre cluster randomised trial carried out in NHS hospitals and NHS/voluntary sector hospices in the United Kingdom. Twelve sites (four hospitals, eight hospices) will participate in the feasibility study and will be randomised to either standard treatment A or standard treatment B. The Surrey Clinical Trials Unit, Surrey CRC, University of Surrey will be responsible for the cluster randomisation, trial management, data management and analysis.

The trial will assess the feasibility of a multi-centre RCT by identifying and addressing the methodological and ethical issues raised. It will provide primary and secondary endpoint data in order to design an appropriately powered RCT.

The trial has been funded by the Research for Patient Benefit Programme of the National Institute of Health Research (National Health Service) and has received a favourable ethical opinion from the London-Bromley National Research Ethics Service (NRES) Committee (reference 14/LO/1543), and the University of Surrey Research Ethics Committee. The trial, current protocol, version 5, 23 February 2015, is registered on ClinicalTrials.gov NCT02344927 (registered 4 June 2014).

Full informed written consent will be obtained from all patients, or their designated consultee, prior to enrolment in the trial.

### Oversight of trial sites

Study sites will be required to develop a cluster representation mechanism (CRM) to represent the interests of the cluster (and the individuals within the cluster). The CRM has the same rights as an individual patient in a normal randomised trial; the CRM has the right to withdraw the cluster from the study if it decides that the study is no longer in the interests of the cluster. The CRM includes a Study Gatekeeper (who is responsible for the cluster as a whole, permits the cluster taking part in the study, and monitors the continued involvement of the cluster in the study, for example, with a senior clinician) and a Study Guardian (who is responsible for the individuals in the cluster, permits individuals to take part in the study, and monitors the continued involvement of individuals in the study, for example, a senior nurse). The CRM will be independent of the research team, and will work to a formal document that describes the role of the CRM.

### Inclusion/exclusion criteria for participants and recruitment

Up to 200 patients will be included in the trial. Patients are eligible for inclusion if they have a diagnosis of cancer; are aged 18 years or above; have an estimated prognosis of ≤ 1 week; and are unable to maintain sufficient oral intake (1 L per day, measured/estimated). Patients will be excluded if they are clinically dehydrated; have a relevant advance directive to refuse treatment; have a clinical indication for clinically assisted hydration (for example, hypercalcaemia); have a clinical contra-indication to clinically assisted hydration (for example, cardiac failure); have a clinical contra-indication to peripheral cannulation; are already being administered intravenous fluids/subcutaneous fluids/total parenteral nutrition (TPN)/enteral feeding or fluids; have symptoms of delirium at the time of consent or have experienced symptoms of delirium in the previous 24 hours; or are likely to be transferred to another setting for endof-life care (for example, home, hospice).

Potential participants will be highlighted to the research team by the clinical team; the research team will then screen the patient for eligibility to enter the study. If the patient is deemed to have capacity by the clinical team, then consent will be sought from the patient in the normal manner by the research team Additional file 1.

If the patient is deemed not to have capacity, then a personal consultee (that is, “someone who has a role in caring for the person who lacks capacity or is interested in that person’s welfare but is not doing so for remuneration or acting in a professional capacity”) will be approached for advice re the patient entering the study [24, 25]. In this study, the personal consultee could be a relation of the person or a friend of the person. The personal consultee will be given an information sheet about the study, given the opportunity to ask questions about the study, and asked whether in their opinion the patient would have any objection to taking part in the study.

If the patient is deemed not to have capacity, and no personal consultee is available, then a nominated consultee will be approached for advice re the patient entering the study [24, 25]. In this study, the nominated consultee will be the Study Guardian (who is independent of the research team).

### Interventions

The interventions utilised within this trial represent current standards of care within clinical practice. Sites will be randomised to either ’standard intervention arm A‘ or ’standard intervention arm B‘, and this will become the standard of care within the site for the duration of this trial.

Standard intervention arm A involves:

- Continuance of oral intake (if appropriate)
- Regular ’mouth care’
- Standard management of pain and other symptoms in the terminal phase

Standard intervention arm B involves:

- Continuance of oral intake (if appropriate)
- Regular ’mouth care’
- Standard management of pain and other symptoms in the terminal phase
- Clinically assisted hydration, that is, parenteral fluids

Mouth care should be performed at least every four hours, and should correspond to the participating site’s policy/procedures for oral care in the terminal phase. Mouth care should be discontinued if it causes distress/discomfort to the patient.

The parenteral fluids may be administered either intravenously or subcutaneously at the discretion of the medical and nursing team [26]. The type of fluid to be administered is dextrose saline (4 % dextrose, 0.18 % sodium chloride), and the volume to be administered will be dependent on the patient's weight [27] Table 1.

### Study procedures

During the treatment period patients will be reviewed at least every four hours, and an assessment made as to whether or not certain symptoms are present, that is, hyperactive delirium (‘terminal agitation‘), pain, excess respiratory secretions (‘death rattle‘), nausea and vomiting, dyspnoea and urinary continence. Agitation will be given a score (see below), but the other symptoms will simply be recorded as present or absent. In addition, changes in regular medications, use of as required (PRN) medications, oral intake, urinary continence, and (if appropriate) parenteral fluids administered, adverse events relating to parenteral fluids and requirements for re-cannulation will also be noted. Data will be obtained from the participant’s observation chart and the participant’s drug chart(s); these documents are considered source documents in this study.

At the end of the study the researcher will record the participant’s outcome, including the date of death/withdrawal, and the reason for withdrawal (if appropriate). The end of study occurs when an individual participant either: a) survives for ≥ 14 days; b) dies (expected outcome); or c) is withdrawn from the study Table 2.

### Outcome measures

#### Observation chart data

The primary outcome will be the frequency of hyperactive delirium (‘terminal agitation‘), assessed using the modified Richmond Agitation and Sedation Scale (mRASS) [28]. The mRASS is a numerical scale that includes four levels of agitation: +1 = restless; +2 = agitated; +3 = very agitated; and +4 = combative. It should be noted that patients with pain/other problems may appear agitated, and so a diagnosis of hyperactive delirium (terminal agitation) must be validated by the clinical team’s use of an appropriate anti-psychotic or sedative drug to treat delirium (terminal agitation).

Other, secondary endpoints will be obtained through the observation chart, including oral intake, problems relating to parenteral fluids and any change in CAH status.

#### Process evaluation

A process evaluation will be undertaken in parallel to the feasibility study using a framework developed for cluster randomised trials [29] (based on the so-called RE-AIM framework [30]): data will be derived from routine research governance/trial monitoring (quantitative data) and additional/independent assessments (quantitative data, qualitative data). Thus, Principal Investigators from all units will be asked to complete a specifically developed questionnaire about the study processes at 1, 3 and 12 months after the start of the study (primarily quantitative data). In addition, clinical staff from 50 % of the units (randomly selected) will be asked to participate in focus groups about the study processes at 3–6 months after the start of the study (qualitative data).

### Data management

Investigator site staff will enter trial data into a paper CRF. The paper CRF is an Annotated Study Book (printed copy of the study eCRF) that has been reviewed by key trial staff including the Chief Investigator, Study Statistician and Trial Project Manager. Once the monitor has visited the site and completed the monitoring visit, completed pages will be stripped and returned to Surrey CTU for data entry into the eCRF. An eCRF will be set up in accordance with the protocol by a Data Manager and reviewed by another Data Manager, with range checks at point of entry and edit checks.

Point of entry checks fire during data entry, to reduce data entry errors. Range and edit checks will be examined by a Data Manager and queries generated as appropriate. Queries raised will be tracked by sending queries from and receiving answers to a Data Management mailbox. Surrey CRC will then update as appropriate.

### Statistical consideration

#### Sample size

Twelve sites (four hospitals, eight hospices) will be involved in the feasibility study, and these will be randomised to either standard intervention A or standard intervention B. There will be a separate randomisation process for the hospitals and the hospices. The target is to recruit 200 participants from the 12 sites within a period of one year; the end of the trial will occur when either 200 participants have been recruited, or the trial has been ongoing for one year (and inadequate numbers of participants have been recruited).

#### Analysis

A ’case‘ of hyperactive delirium is considered to be a participant that scores +2 to +4 on the mRASS [28], and is treated by the clinical team with an appropriate anti-psychotic or sedative drug to treat hyperactive delirium (terminal agitation). Participants who do not fall into this category will be considered as ’non-case‘. This dichotomous outcome will be used as the primary endpoint for the analysis.

The analysis evaluating the difference in proportions of participants experiencing hyperactive delirium between the two interventions will be a logistic regression with the occurrence of hyperactive delirium as the dependent variable and interventions (A or B), cluster and cluster type (hospital or hospice) as explanatory variables. The analysis will be based on the intention-to-treat (ITT) population, and will furnish estimates of the proportion of subjects suffering from hyperactive delirium for each intervention together with an estimate of the coefficient of variation of true proportions between clusters within each intervention arm. The latter of these estimates is an important requirement for determining the sample size aspect of a future cluster randomised study designed with adequate power to evaluate the impact of the intervention including CAH. It is judged that recruitment of 200 participants in 12 clusters in the feasibility study will provide a realistic estimate.

The logistic regression analysis will allow the testing of the hypothesis of no difference in the proportion of hyperactive delirium experienced between groups treated by either intervention. The intervention including CAH will be considered an improvement on the other intervention, in this and any future definitive study, only if the proportion of hyperactive delirium sufferers is reduced by at least 20 %.

It is considered unlikely that this statistical analysis of the feasibility study will be able to test this hypothesis with sufficient power, but its results, together with information gained from other aspects of the study management, will be used to design a definitive study.

From a statistical perspective, the estimates of the proportion of subjects suffering hyperactive delirium for each intervention, and the coefficient of variation of true proportions between clusters within each intervention, obtained from the feasibility study, will be used to determine the number of clusters and sample size necessary to detect a difference of 20 % in the proportion of hyperactive delirium sufferers between the interventions with 80 % power using a two-sided 5 % significance level. This calculation will form part of the design of the definitive study.

Should the number of clusters and sample size in the feasibility study retrospectively be found to provide the necessary power to test the hypothesis, the statistician will provide this information to the study team as input into their decision as to whether a definitive study is required.

### Governance

#### Safety reporting

All adverse events (AEs) and serious adverse events (SAEs) will be documented in the CRF, and reviewed by the Chief Investigator and Trial Monitoring Committee (TMC). As the study is being undertaken in patients in the last days of life, the progression of existing problems and the development of routine end-oflife problems (for example, hyperactive delirium/’terminal agitation‘, excess respiratory secretions/’death rattle‘) are not considered to be SAEs.

#### Monitoring

The Surrey CTU, Surrey CRC were responsible for the management and coordination of the clinical trial, including communication of important protocol modifications to trial investigators and trial sites. The Surrey CTU will monitor trial sites on a regular basis to ensure adherence to Good Clinical Practice, and especially compliance with the protocol. The monitor will inspect the site file and will check the recruitment/screening log and source documents.

#### Trial Monitoring Committee

The study will have a Trial Monitoring Committee (TMC), which will consist of an independent Chairperson (Consultant in Palliative Medicine), the Chief Investigator, the Clinical Trial Unit Lead, the Lead Research Nurse, the Statisticians, the Ethical Advisor, patient/carer representatives and one of the Principal Investigators. The TMC will meet approximately once a quarter (until the end-of-study report has been completed). The TMC will review all aspects of the study, particularly any safety issues.

#### Confidentiality

The study will conform to the Data Protection Act (and related legislation); all data will be treated as confidential, and data will be anonymised prior to removal from the study site.

#### Publication and dissemination

At the end of the study, a study report will be written and submitted to the funder, the Sponsor, the REC and the Principal Investigators.

The results of the study will be published in an appropriate medical journal and presented at appropriate medical conferences. The main publication will be authored by the Chief Investigator with contribution from the Surrey CTU. Upon completion of the Clinical Trial, or when the Clinical Trial data are adequate (in the Sponsor’s reasonable judgement), an Investigator site may prepare the data deriving from the Clinical Trial for publication. Such data will be submitted to the Sponsor and Chief Investigator for review and comment prior to publication.

## Discussion

The provision of clinically assisted hydration (CAH) at the end of life is one of the most contentious issues in medicine [1, 2], and indeed within the general population [3, 4]. In some instances there is a clear indication for giving CAH (such as for a patient with opioid toxicity), whilst in other instances there is a clear indication for not giving CAH (for example, in a patient with cardiac failure). Currently, however, it is impossible to make a best interests decision for the majority of patients (due to a lack of evidence). Hence, there is an urgent need/ethical necessity to undertake research to define the role of CAH in the last days of life (and indeed the role of mouth care in the last days of life).

The study interventions to be used in the trial are routinely used in clinical practice, and so all of the patients will receive a ’standard intervention‘ (and not an experimental treatment, or a placebo treatment). Indeed any patients who require a specific intervention or for whom CAH is contra-indicated will not be considered eligible for the trial.

The study team is mindful that the trial involves patients in the last week of life, and it is anticipated that many of the potential participants will be unable to provide informed consent (due to impaired cognition/impaired consciousness). As the study is not a Clinical Trial of an Investigational Medicinal Product (CTIMP), the study comes under the remit of the Mental Capacity Act [24, 25]. Consent will be sought from the patient (whenever possible), or advice sought from a ’personal consultee‘ (when the patient is unable to provide consent), or advice from a ’nominated consultee‘ (when the patient is unable to provide consent, and there is no personal consultee). In this study the personal consultee will be a relation or friend of the patient, and the nominated consultee will be the Study Guardian. It is hoped that this trial will allow a definitive study to be performed of the utility/role of CAH in patients with cancer in the last days of life.

## Trial status

Patient recruitment commenced on 20 February 2015.

## Additional file

Additional file 1:
**Patient information sheet. Information regarding the study to enable patients or their designee to make a decision as to whether they wish to participate in the trial.** (DOCX 63 kb)

## Abbreviations

CAH: Clinically assisted hydration
CTIMP: Clinical trial of investigational medicinal product
Surrey CRC: Surrey clinical research centre
CTU: Clinical trials unit
