# Supplementary material for: Alternative forms of hydration in patients with cancer in the last days of life: study protocol for a randomised controlled trial
Source: Trials. 2015 Oct 14;16:464. doi: 10.1186/s13063-015-0988-3 (PMC4607172; doi:10.1186/s13063-015-0988-3)
Supplement: Additional file 1: — Patient information sheet. Information regarding the study to enable patients or their designee to make a decision as to whether they wish to participate in the trial. (DOCX 63 kb) [file 13063_2015_988_MOESM1_ESM.docx]

**INFORMATION ABOUT THE RESEARCH**

**Study title: A cluster randomised trial of alternative forms of hydration in cancer patients in the last days of life**

**Protocol number: 5**

**Protocol date: 23^th^ Feb 2015**

*We would like to invite you to take part in a research study. Before you decide we would like you to understand why the research is being done and what it would involve for you.* ***One of our team will go through the information sheet with you and answer any questions you have.*** *We’d suggest this should take about 15 minutes. Talk to others about the study if you wish.*

*(Part 1 tells you the purpose of this study and what will happen to you if you take part. Part 2 gives you more detailed information about the conduct of the study). Ask us if there is anything that is not clear.*

**Part 1**

**What is the purpose of the study?**

As people get closer to death, they tend to eat and drink less than beforehand. When this occurs, it is often unclear to doctors and nurses what they should do for the patient. In some hospitals and hospices the standard treatment is to encourage / help the patient to drink, and to give the patient regular “mouth care”. Mouth care usually involves someone applying water or an artificial saliva product to the inside of the mouth (and a lubricant to the lips). However, in other hospitals and hospices the standard treatment is to give fluid by a drip either into a vein or under the skin (as well as to encourage / help the patient to drink, and to give the patient regular mouth care).

The reason for this difference in approach is that there is no research to guide doctors and nurses as to what they should do for the majority of patients. However, there are some instances where it is clear that the patient should be given fluid by a drip as the potential benefit appears to outweigh the potential harm. Similarly, there are some instances where it is clear that the patient should not be given fluid by a drip as the potential harm appears to outweigh the potential benefit, e.g. in a patient with fluid retention (swollen legs / arms). The aim of this research study is to provide evidence to allow doctors and nurses to make the right decision in the majority of patients.

The research study is being funded by the Research for Patient Benefit Programme (a part of the National Health Service). The study is being conducted in a number of different hospitals and hospices, because the palliative care teams in these units want to ensure that all patients receive the best end-of-life care available, and that all treatments given at the end-of-life are effective (i.e. they do work), and are well tolerated (i.e. they don’t cause side effects). Currently, some of these hospitals and hospices usually give fluid by a drip, whilst other hospitals and hospices usually do not give fluid by a drip (but do encourage / help the patient to drink, and to give the patient regular mouth care).

For the purpose of the study, all of the hospitals and hospices will adopt a standard treatment for hydration of patients, but the treatment that they use will be decided by chance (randomly): 50% hospitals and hospices will be giving fluid by a drip, and 50% hospitals and hospices will be encouraging / helping the patient to drink, and giving the patient regular mouth care. In this hospital / hospice, the standard treatment is fluids by a drip, and we only use drinking & regular mouth care alone if there is a very good reason to do so. Your palliative care / clinical team have decided that you should receive the standard treatment at the moment, but will continue to monitor your condition may change this decision based on their clinical judgement. The clinical team, and not the research team, will have the final say in this matter.

The purpose of the study is to see whether the problems experienced at the end-of-life are different in the two groups, and how well patients tolerate the different treatments. The care you will receive will be the same as if you were not in the study, and the study will not involve any more questions, observations, or investigations. The doctors and nurses routinely collect information on all patients; this information will be shared with the research team (and analysed by the University of Surrey). The researcher team will collect the information from your observation chart, drug chart, and medical and nursing notes: the research team will have no direct contact with you during the actual study.

**Why have we been chosen?**

The reason we have approached you about the study is that you seem to be approaching the end-of-life, and are drinking less than normal, or are expected to soon be drinking less than normal. Also, there appears to be no reason not to give you the standard treatment that is used in this hospital / hospice (i.e. fluids by a drip). The palliative care team referred you to the research team, as they felt you may be willing to participate in this study.

We are intending to enrol 200 similar patients to the study from twelve hospitals and hospices in the United Kingdom.

**Do I have to take part?**

It is up to you to decide to join the study. We will describe the study and go through this information sheet. If you agree to take part, we will ask you to sign a consent form. A copy of the consent and this information sheet will be given to you to keep. You are free to withdraw at any time, without giving a reason. This would not affect the standard of care you receive.

**What will happen to me if I take part?**

If you decide to take part in the study, then the care you will receive will be the same as if you were not in the study: you will receive fluids by a drip, as this is the standard treatment in this hospital / hospice.

The nursing staff will assess you at least every four hours, and record any symptoms or other problems you may experience. If you do develop any symptoms or other problems, then these will be treated in the normal way. The nursing staff will record any drugs or other treatments you may receive, and any problems relating to fluids by a drip. These observations are done routinely, and there will be no more observations than normal.

All of the care will be done by your normal doctors and nurse, and you will have no contact with the research team during the actual study. The research team will collect information about your symptoms, other problems, drugs given, other treatments given, and any problems relating to fluids by a drip. This information will be anonymised (so that your identity is protected), and then sent to the University of Surrey for analysis.

**What do I have to do?**

If you decide to take part in the study, then you will need to sign a consent form to say that you agree to take part in the study, and that you agree for the research team to have access to / use the routine information collected by your clinical team (doctors and nurses). Otherwise, there is nothing for you to do.

If you decide not to take part in the study, then your care will be the same as if you take part in the study, but the research team will not have access to / use the routine information collected by your clinical team.

**What are the alternatives for treatment?**

The standard treatment in this hospital / hospice is fluids by a drip. Some of the other hospitals and hospices in the study will have a different standard treatment, i.e. drinking & regular mouth care alone.

**What are the possible disadvantages and risks of taking part?**

If you decide to take part in the study, then the care you will receive will be the same as if you were not in the study, and so there are no additional risks (or disadvantages).

Mouth care is sometimes uncomfortable, and if this is the case then the doctors and nurses may decide to stop the treatment altogether.

Inserting the drip can be uncomfortable, and some people may experience bruising / soreness at the site of the drip. Some patients may also experience fluid retention, which may cause swelling of the arms and legs, and sometimes shortness of breath (due to fluid on the lungs). If this is the case then the doctors and nurses may decide to stop this treatment altogether.

**What are the possible benefits of taking part?**

We cannot promise the study will help you but the information we get from this study will help to improve the treatment of people with advanced cancer in the future.

**What if there is a problem?**

Any complaints about the way you have been dealt with during the study or any possible harm you might suffer will be addressed. The detailed information on this is given in Part 2.

**Will my taking part in the study be kept confidential?**

Yes. We will follow ethical and legal practice and all information about you will be handled in confidence. The details are included in Part 2.

*If the information in Part 1 has interested you and you are considering participation, please read the additional information in Part 2 before making any decision*

**Part 2**

**What if relevant information becomes available?**

Sometimes we get new information about the treatment being studied. If this happens, your researcher doctor might consider you should withdraw from the study. He / she will explain the reasons and arrange for your care to continue.

**What will happen if don’t want to carry on with the study?**

You are free to withdraw from the study at any stage, and you do not have to give a reason for withdrawing from the study.

If you do withdraw from the study, then your care will not be affected. The research team will continue to use the clinical information collected up to the time you withdraw from the study, but the research team will not be allowed to use any clinical information collected after the time you withdraw from the study.

**What if there is a problem**

If you have a concern about any aspect of this study, you should ask to speak to the researchers who will do their best to answer your questions.

*Researcher name: Dr Andrew Davies – Consultant in Palliative Medicine*

*Contact phone number: 01483 571122 ext. 2043*

If you remain unhappy and wish to complain formally, you are free to do this through the hospital Patient Advice and Liaison Service / hospice complaints procedure. Details can be obtained from

*Name:* *Patient Advice and Liaison Service*

*Contact phone number:* *01483 402757*

*Email:* rsc-tr.PALS@nhs.net

In the event that something does go wrong and you are harmed during the research and this is due to someone’s negligence then you may have grounds for a legal action for compensation against the hospital / hospice but you may have to pay legal costs. The normal National Health Service / hospice complaints mechanisms will still be available to you.

**Will my taking part in this study be kept confidential?**

All information which is collected about you during the course of the research study will be kept strictly confidential, and any information about you which leaves the hospital / hospice will have your name and address removed so that you cannot be recognised.

If you join the study, some parts of your medical records and data collected for the study will be looked at by authorised persons from the University of Surrey (who are organising the research). They may also be looked at by authorised people to check the study is being carried out correctly. All will have a duty of confidentiality to you as a research participant and we will do our best to meet this duty.

**What will happen to the results of the research study?**

The results of the study will be fed back to the Research for Patient Benefit Programme (National Health Service), and then presented at medical meetings, and published in medical journals

No patient will be identifiable in any reports, presentations, or publications of the study.

**Who is organising and funding the research?**

The research study is being funded by the Research for Patient Benefit Programme (National Health Service), and is being organised (“sponsored”) by the University of Surrey.

The hospital / hospice / any individual will not be paid for including you in the study.

**Who has reviewed the study?**

All research in the NHS is looked at by independent group of people, called a Research Ethics Committee, to protect your interests. This study has been reviewed and given favourable opinion by the London- Bromley Research Ethics Committee.

The study has also been reviewed / approved by the Research for Patient Benefit Programme (National Health Service), and by the University of Surrey.

**Further information and contact details**

If you would like further information about the study, then please contact the following members of the research team:

*Name Principal Investigator: Dr Andrew Davies – Consultant in Palliative Medicine*

*Contact phone number: 01483 571122 ext. 2043*

*Email:* *adavies12@nhs.net*

*Name Research Nurse (if applicable): Alison Buchanan*

*Contact phone number:01483 571122 ext. 2777*

*Email:abuchanan1@nhs.net*
